# Sprouting Time Affects Sorghum (*Sorghum bicolor* [L.] Moench) Functionality and Bread-Baking Performance

**DOI:** 10.3390/foods10102285

**Published:** 2021-09-27

**Authors:** Gaetano Cardone, Rubina Rumler, Sofia Speranza, Alessandra Marti, Regine Schönlechner

**Affiliations:** 1 Department of Food, Environmental and Nutritional Sciences (DeFENS), University of Milan, Via G. Celoria 2, 20133 Milan, Italy; gaetano.cardone@unimi.it; 2 Department of Food Science and Technology, Institute of Food Technology, University of Natural Resources and Life Sciences, Muthgasse 18, A-1190 Vienna, Austria; rubina.rumler@boku.ac.at (R.R.); sofia.speranza@boku.ac.at (S.S.); regine.schoenlechner@boku.ac.at (R.S.)

**Keywords:** sorghum, germination, flour functionality, rheology, bread, starch digestibility, protein digestibility

## Abstract

Despite being considered a climate-resilient crop, sorghum is still underutilized in food processing because of the limited starch and protein functionality. For this reason, the objective of this study was to investigate the effect of sprouting time on sorghum functional properties and the possibility to exploit sprouted sorghum in bread making. In this context, red sorghum was sprouted for 24, 36, 48, 72, and 96 h at 27 °C. Sprouting time did not strongly affect the sorghum composition in terms of total starch, fiber, and protein contents. On the other hand, the developed proteolytic activity had a positive effect on oil-absorption capacity, pasting, and gelation properties. Conversely, the increased α-amylase activity in sprouted samples (≥36 h) altered starch functionality. As regards sorghum-enriched bread, the blends containing 48 h-sprouted sorghum showed high specific volume and low crumb firmness. In addition, enrichment in sprouted sorghum increased both the in vitro protein digestibility and the slowly digestible starch fraction of bread. Overall, this study showed that 48 h-sprouted sorghum enhanced the bread-making performance of wheat-based products.

## 1. Introduction

Although sorghum (*Sorghum bicolor* [L.] Moench) is a staple food for the populations of the sub-Saharan regions, it is becoming an interesting ingredient in those formulations which are typical of the Western countries [1,2,3,4,5,6]. Sorghum has been defined as the “crop of the future” thanks to its high resistance to semi-arid soils and its low water requirements [7]. In addition to the agronomic traits, from a nutritional standpoint, sorghum is a good source of dietary fiber, vitamins, minerals, and phenolic compounds [8]. Moreover, being a gluten-free cereal, sorghum is also suitable for the diet of people suffering from celiac disease. On the other hand, sorghum is characterized by low protein digestibility, due to the presence of protein bodies formed by kafirins (i.e., storage proteins with high hydrophobicity) stabilized by disulphide bonds [9]. In addition, these structures form a tight starch–protein matrix that leads not only to a decrease in starch and protein digestibility [9,10]**,** but also to a decrease in starch gelatinization properties [11,12]. This is critical from a technological standpoint because starch pasting and gelation properties represent a key aspect in food products by affecting their final characteristics such as viscosity, structure, and texture. For these reasons, the use of sorghum in food production is still limited. As regards wheat-based bread, the presence of sorghum (from 10%) decreases bread volume and increases dry mouthfeel and crumb firmness [13]. For this reason, sorghum should be treated in a way that improves its functionality, to obtain baked goods with satisfactory attributes for consumers (i.e., high volume and crumb firmness). In this context, sprouting has been proposed as a useful bio-process to modify the structure of sorghum, enhancing its functionality, in terms of oil absorption capacity, emulsion, and foam stability [14,15,16,17,18,19]. At the same time, sprouting is associated with a decrease in starch swelling and pasting properties, and cross-linked kafirins [4,14,16,17,18,19,20,21], with a positive effect on the in vitro protein digestibility [18,19,21]. Furthermore, sprouting is related to an increase in mineral bioavailability, polyphenol content, and antioxidant capacity, as well as to a decrease in antinutritional factors (e.g., condensed tannins and trypsin inhibitors) [22,23]. Although several researchers have already investigated the effects of sprouting on chemical composition and functional properties of sorghum [4,14,16,17,18,19,20,21], to the best of our knowledge, the relation between these changes and the properties of sprouted sorghum-enriched bread have not been studied yet.

Considering the aspects reported above, the purpose of this research was to assess the relationship between the changes in flour functionality—induced by sprouting time—and bread-making performance of bread enriched with sprouted sorghum.

## 2. Materials and Methods

### 2.1. Materials

De-husked and tannin-free sorghum (Sorghum *bicolor* [L.] Moench; Armorik *cv.*) was purchased from Caj. Stobl Naturmühle (Linz-Ebelsberg, Austria). Grains were grown and harvested on an experimental field in Hörsching (Oberösterreich, Austria) in 2019. Six aliquots (1 kg each) of grains were sprouted in a climate chamber (Model 60/rW, MANZ Backtechnik GmbH, Creglingen, Germany). Specifically, seeds were soaked (1:3 *w/w*) for 16 h at 27 ± 2 °C (90% Relative Humidity, RH) and sprouted for 24, 36, 48, 72, and 96 h, at 27 ± 2 °C (90% RH). After sprouting, seeds were dried at 50 °C for 9 h (Self Cooking Center, Rational International AG, Landsberg am Lech, Germany). Untreated sorghum was used as control (CTRL). All samples were ground by means of the Retsch**^®^** ZM 200 Mill (Verder Scientific GmbH & Co. KG, Golling, Austria) equipped with a 0.5 mm screen. Wholegrain flours were stored at 4 °C for 7 days before using.

Commercial wheat flour (Fini’s Feinstes; protein content: 14 g/100 g of flour; W = 290 × 10**^−^**^4^ J) was used as the base for sorghum replacement at 20% level.

### 2.2. Chemical Composition and Enzymatic Activity of Sorghum Flour

Moisture [24], total [25] and damaged [26] starch, protein [27], total, insoluble, and soluble dietary fiber [28] contents, as well as α-amylase [29] and protease [30] activities, were determined according to the official methods. Simple sugars (i.e., maltose, sucrose, and D-glucose) were quantified by means of enzymatic kit (K-MASUG; NEOGEN/Megazyme, Lansing, MI, USA).

### 2.3. Sorghum Flour Functionality

#### 2.3.1. Water (WAC) and Oil (OAC) Absorption Capacity

WAC and OAC were evaluated following the method reported by Marchini et al. [20]. Briefly, 1 g of flour was weighted in a 50 mL plastic tube and vortexed for 1 min with water or sunflower oil (1:10 *w/v*), respectively. Tubes were left to decant at 21 °C for 30 min. Finally, samples were centrifuged at 4000*×g* for 20 min and the supernatants were discarded. WAC and OAC were expressed as g of water or oil absorbed per 100 g of flour d.b.

#### 2.3.2. Swelling Power (Sp) and Pasting Properties

Sp was determined according to Zhang and Hamaker [31]. Pasting properties were evaluated by using the Rapid Visco Analyzer (RVA**^®^** 4500; PerkinElmer, Inc., Spokane, WA, USA), by dispersing 3.5 g (14% dry matter) of flour in 25 g of distilled water or silver nitrate solution (1 mM; AgNO_3_)—as a strong α-amylase inhibitor. The temperature profile applied was in accordance with the standard method [32].

### 2.4. Protein Features

#### 2.4.1. Kafirin Extraction and Electrophoresis Analysis (SDS-PAGE)

The extraction of kafirins and their electrophoretic profiles (i.e., SDS-PAGE) were carried out according to the method proposed by Carter and Reck [33] with slight modifications undertaken by Espinosa-Ramírez and Serna-Saldívar [34].

The SDS-PAGE was performed by using a Mini-Protean II cell (Bio-Rad) at 200 V by using 4–15% Mini-PROTEAN**^®^** TGX™ precast gels. Extracted kafirins were dissolved in the SDS sample buffer (62.5 mM Tris/HCl, 2% SDS, 25% glycerol, 0.01% bromophenol blue). Electrophoretic analysis was carried out also under reducing conditions by adding 1.4% (*v*/*v*) of β-mercaptoethanol at SDS sample buffer. After the samples were boiled for 5 min, 10 µg of protein were loaded. A broad unstained protein ladders (10**–**250 kDa) was used (BioRad, Richmond, CA, USA). Protein bands were fixed by using 10% (*v*/*v*) acetic acid solution (for 30 min), stained with brilliant blue G250 (Sigma-Aldrich, St. Louis, MO, USA) (for 30 min) and distained with 20% ethanol and 10% acetic acid solution (for 15 h).

#### 2.4.2. Protein Solubility and Thiol Accessibility

Protein solubility under different conditions (i.e., native, reducing and/or denaturing) and thiol accessibility were assessed by following the methods previously reported by Marengo et al. [4].

### 2.5. Sorghum–Wheat Blend Functionality

The gluten aggregation kinetics of blends were evaluated by means of the GlutoPeak (Brabender GmbH & Co., Duisburg, Germany) test, according to Suárez-Estrella et al. [35], by using 10 g of distilled water instead of 9 g.

The mixing properties of the blends were determined according to the ICC official method (ICC 115/1) [36], by using the Farinograph (Brabender GmbH & Co., Duisburg, Germany) device, equipped with 50 g mixing bowl.

### 2.6. Bread Making

Bread doughs were prepared according to the ICC official method (ICC 131) [37]. Specifically, bread doughs were made as follows: flour, fresh baker’s yeast (2% of flour; Hagold Hefe GmbH, Austria), salt (2% of flour; Salinen Co., Ebensee, Austria), and tap water (65%) at 25 °C. Flour and salt were mixed for 1 min using an automatic mixer (Varimixer Teddy, Varimixer, Denmark). After that, yeast was dissolved in water and added into the mixing bowl. Dough was kneaded for 6 min and left to rest in a leavening chamber (BS60/3, Manz Co., Creglingen-Münster, Germany) for 30 min at 30 °C (85% RH). The dough was divided into three sub-samples (300 g each), shaped, and put into the baking pans (length: 12.5 cm; width: 6 cm; height: 5 cm). After that, sub-samples were left to proof for 50 min (30 °C and 85% RH) and then baked for 35 min at 180 °C (BS60/3, Manz Co., Creglingen-Münster, Germany), with vapor injection.

### 2.7. Bread Properties

Bread volume (mL) was evaluated by means of the VolScan Profiler (Stable Micro Systems, Surrey, UK) and bread specific volume (mL/g) was calculated through the volume/mass ratio. Crumb firmness was determined according to the AACC official method [38]. Crumb color profile was determined through the digital colorimeter (Digital Color Meter, Apple Inc, Cupertino, USA) and expressed according to the CIE-L × a × b× color space.

In vitro starch and protein digestibility of bread was performed by following the Englyst [39] and Hsu et al. [40] methods, respectively. *Streptomyces griseus* (Type XIV, ≥3.5 units/mg solid, Merk) was used instead of protease from porcine intestinal, as suggested by Vilakati et al. [41].

### 2.8. Statistical Analysis

All analyses were replicated three times. Three baking tests were performed, and three loaves were obtained from each test (*n* = 9). Bread volume was measured from each loaf and the crumb firmness was evaluated on the three central bread slices of each bread, for a total of 27 measurements. Crumb color profile was replicated three times on the central slice from each loaf. In vitro starch and protein digestibility were determined on one slice from each bread of each baking trial, for a total of 9 slices. All data were subjected to analysis of variance (one-way ANOVA; α = 0.05) by using Statgraphics XV version 15.1.02 (StatPoint Inc., Warrenton, VA, USA). When a factor resulted significantly different, the difference was determined through the Tukey HSD test. In addition, data were processed by Principal Component Analysis (PCA) by using Statgraphic Plus v. 5.1. (StatPoint Inc., Warrenton, VA, USA).

## 3. Results

### 3.1. Chemical Composition and Enzymatic Activities

Total starch and protein contents were slightly affected by sprouting time, although hydrolytic activities (i.e., α-amylase and protease) significantly increased during the process (Table 1).

Similar results were found in studies on sprouted wheat that applied similar conditions to those reported here [42,43]. On the other hand, Elmaki et al. [18] reported that total starch content decreased from 30% to 50%, when sorghum was sprouted at 30 °C from 24 to 72 h, whereas Marchini et al. [15] reported a smaller decrease in starch content (by about 5%) when sorghum was sprouted at 25 °C for 72 h. Different results among the studies could be related to differences in varieties, as well as in sprouting conditions.

In contrast, the sprouting process did not strongly affect the total dietary fiber of sorghum (Table 1), in accordance with Marchini et al. [15]. The changes in the insoluble and soluble fiber could be related to the fact that fiber components do not precipitate upon ethanol addition but remain in solution, resulting in an underestimation of the soluble fraction [44]. On the other hand, damaged starch (~22%), maltose (~170%), and glucose (~96%) increased starting from 48 h of sprouting (Table 1), due to the increase in α-amylase activity (Table 1). Indeed, simple sugars are necessary to provide energy for the development of the new plant [18]. In contrast**,** sucrose decreased during the early stages of sprouting as seeds use it as a primary source of energy [45], whereas its increase after 72 h of sprouting was attributed to the higher enzymatic activity synthesizing sucrose [46].

As regards the enzymatic activities, sprouting (at all times) increased both amylolytic and proteolytic activities. In particular, the increase in α-amylase activity was more intense than proteolytic ones (~37- vs. ~2-folds).

### 3.2. Functional Properties

Sprouting caused a significant decrease (~5%) in WAC from 48 h, while OAC increased (~20%) from 24 h (Table 2), as an effect of amylase and protease activity (Table 1). However, at 72 h and 96 h, the sprouted sample did not show differences with CTRL in terms of OAC. In contrast, sprouting for 96 h significantly decreased Sp, regardless of the temperature considered (85 and 100 °C).

**Figure 1:**
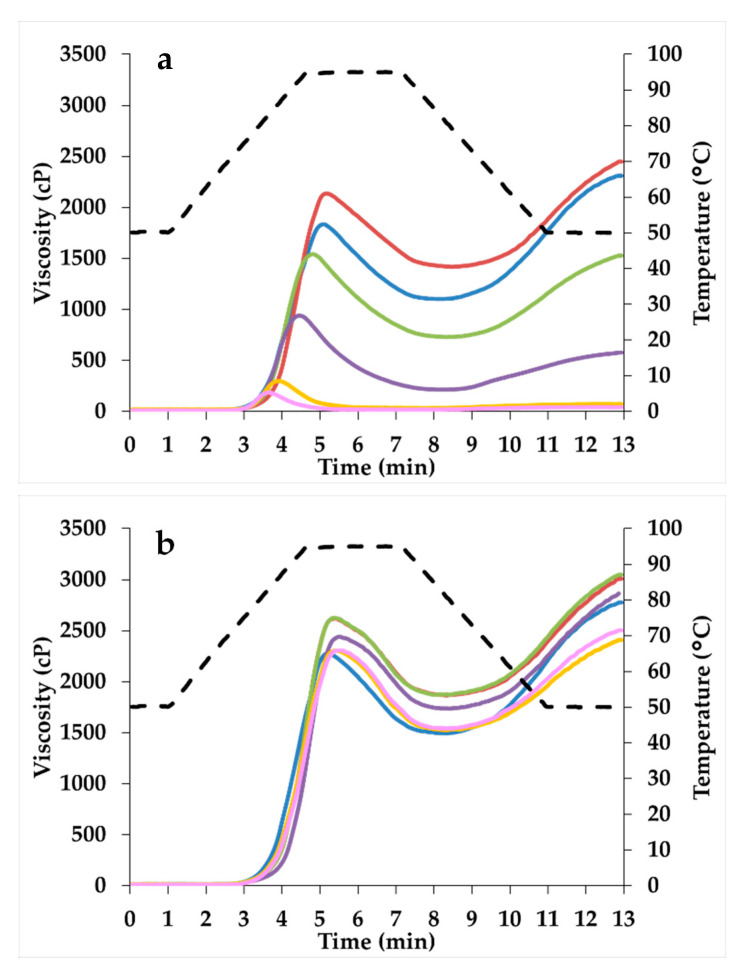
Profiles of pasting and gelation properties of flours from unsprouted (CTRL) and sprouted sorghum at different times (24 h, 36 h, 48 h, 72 h, and 96 h), by using (**a**) water or (**b**) AgNO_3_, as solvent. CTRL: blue line; 24 h: red line; 36 h: green line; 48 h: purple line; 72 h: yellow line; 96 h: pink line.

As regards the starch pasting properties using water as solvent, the 24 h sample showed a higher viscosity value than CTRL (Table 2; Figure 1a). However, as sprouting time increased (>24 h), viscosity decreased, as a consequence of increased α-amylase activity (Table 1). In particular, the 72 h- and 96 h-sprouted samples showed the lowest values throughout the duration of the test (Figure 1a). Moreover, peak temperature increased in the first 36 h of sprouting.

Inhibiting the α-amylase activity during the test—by using silver nitrate as solvent—all the samples showed viscosity values higher or comparable to CTRL (Figure 1b). In particular, samples sprouted up to 36 h showed a significant higher peak and final viscosity compared to CTRL; on the other hand, no significant differences were observed among CTRL and the samples that were sprouted from 48 to 96 h (Figure 1b).

### 3.3. Protein Features

**Figure 2:**
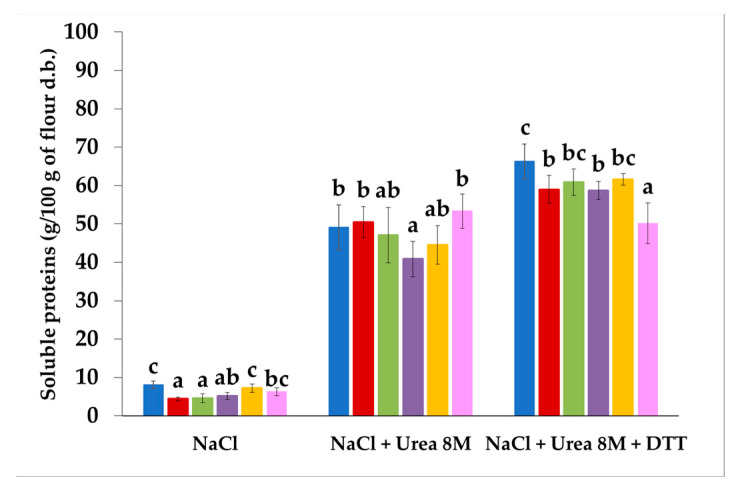
Amount of proteins of unsprouted (CTRL) and sprouted sorghum at different times (24 h, 36 h, 48 h, 72 h, and 96 h), solubilized in various conditions. Different letters in the same condition indicate a significant difference among samples (one-way ANOVA; Tukey HSD test; *p* ≤ 0.05). CTRL: blue bar; 24 h: red bar; 36 h: green bar; 48 h: purple bar; 72 h: yellow bar; 96 h: pink bar.

The content of soluble proteins in the phosphate buffer + NaCl, significantly decreased from 24 h to 48 h of sprouting (~44%) (Figure 2). As the sprouting progressed, the protein solubility increased again, showing no differences with the CTRL anymore. The addition of the chaotropic agent (i.e., Urea 8 M) did not strongly affect the solubility of the proteins stabilized by hydrophobic interactions. In contrast, the content of proteins stabilized by both hydrophobic interactions and disulfide bonds—evaluated by adding DTT—slightly decreased starting from 24 h of sprouting, reaching the minimum value after 96 h.

The content of free accessible thiols underwent a significant decrease during sprouting (1.5 μmol/g of flour d.b. for CTRL and, 1.1, 1.2, 1.1, 1.1 μmol/g of flour d.b. for 24 h-, 36 h-, 48 h-, and 96 h-sprouted samples), except for the 72 h-sprouted sample (1.3 μmol/g of flour d.b). The addition of chaotropic agent, to evaluate the total accessible thiols, led to an increase in their content by about two times (2.6 μmol/g of flour d.b. for CTRL, and 1.8, 2.0, 2.1, 1.9, and 2.7 μmol/g of flour d.b. for 24 h-, 36 h-, 48 h-, 72 h-, and 96 h-sprouted samples, respectively), compared to free accessible ones, regardless of the sprouting time. Interestingly, the 96 h-sprouted sorghum showed a similar value to the CTRL sample for this index.

**Figure 3:**
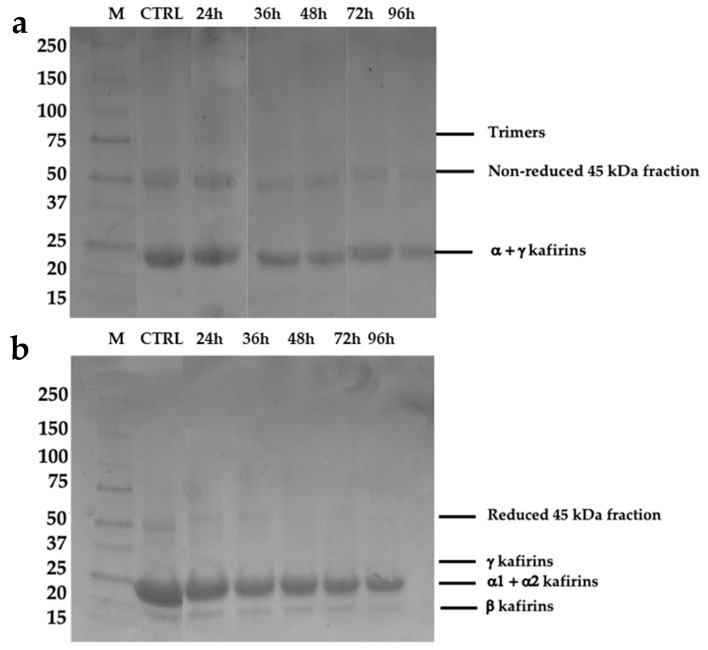
Electrophoretic profile of extracted kafirins in absence (**a**) and in presence (**b**) of reducing agent (β-mercaptoethanol). M: Marker.

The electrophoretic analysis (SDS-PAGE) of kafirins in absence and in presence of reducing agent (β-mercaptoethanol) are shown in Figure 3a,b, respectively.

Under unreduced conditions, samples might be identified into two groups on the base of the band density. Specifically, CTRL and 24 h-sprouted samples, as well as 36 h-, 48 h-, 72 h-, and 96 h-sprouted samples showed the same band density. Moreover, under these conditions, trimers (~75 kDa) and non-reduced fractions (~50 kDa) were found (Figure 3a). In contrast, under reducing conditions, the electrophoretic analysis did not show trimers bands but showed the presence of γ−, α1− + α2−, and β-kafirins at 28–30, 21–23, and 17–18 kDa, respectively (Figure 3b). Since β− and γ-fractions are located on the outer part of the protein bodies, they are the first to be hydrolyzed by proteases during sprouting. Instead, the α1- and α2-fraction of kafirins are present in the inner part of the protein body [11]. The main kafirin fraction was represented by α−kafirins, since their bands are composed of the overlapping of α1− and α2−kafirin subunits. Indeed, these subunits are characterized by low different molecular weight (Figure 3b), and consequently slight different mobility. Moreover, faint bands related to γ−kafirins were shown only in CTRL and dried samples, instead α1−, α2−, and β−kafirins were shown in all samples, even if their density decreased upon sprouting.

### 3.4. Effects of Sprouting on Sorghum–Wheat Blend Functionality

**Figure 4:**
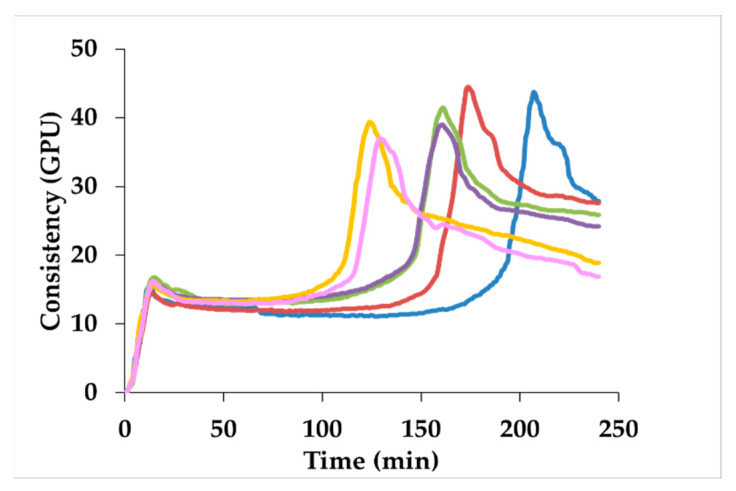
Gluten aggregation profiles of wheat flour containing 20% unsprouted (CTRL) or sprouted sorghum at different times (24 h, 36 h, 48 h, 72 h, and 96 h). CTRL: blue line; 24 h: red line; 36 h: green line; 48 h: purple line; 72 h: yellow line; 96 h: pink line. GPU: GlutoPeak Units.

As regards the gluten aggregation kinetics of composite flours (Figure 4; Table 3), the presence of sprouted samples caused a significant decrease in all the indices considered (Figure 4; Table 3). Specifically, sorghum sprouted from 72 h determined a significant decrease in the maximum torque (~12%) and in the aggregation energy (~63%), while the peak maximum time was already affected starting from 24 h of sprouting (~38%).

**Figure 5:**
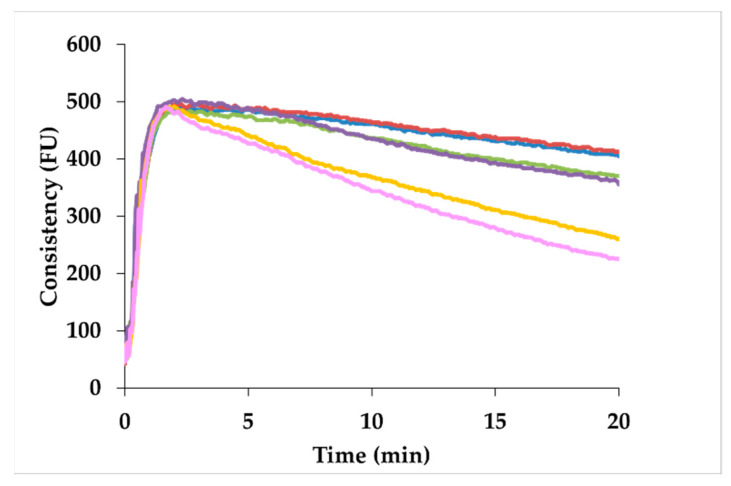
Mixing profiles of wheat flour containing 20% unsprouted (CTRL) or sprouted sorghum at different times (24 h, 36 h, 48 h, 72 h, and 96 h). CTRL: blue line; 24 h: red line; 36 h: green line; 48 h: purple line; 72 h: yellow line; 96 h: pink line. FU: Farinograph Units.

As regards the effects of sprouting time on the mixing properties (Table 3; Figure 5), the presence of sprouted sorghum did not affect the water absorption of dough, but it led to a decrease in both dough development time (~37%) and stability (from ~44% to ~77%), when samples sprouted for 96 and 48 h were used, respectively. The decrease in both indices was an effect of the proteolytic activity developed during sprouting (Table 1). In addition, a great increase in the degree of softening was observed starting from 36 h of sprouting compared to CTRL (Table 3).

### 3.5. Bread Properties

**Figure 6:**
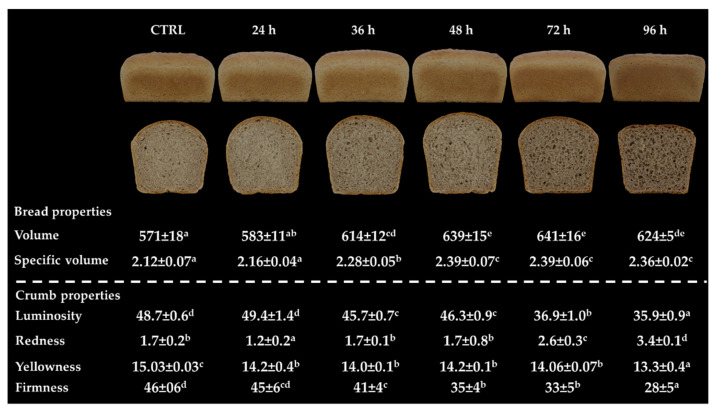
Properties of bread from wheat containing 20% unsprouted (CTRL) or sprouted sorghum at different times (24 h, 36 h, 48 h, 72 h, and 96 h). Different letters in the same row indicate a significant difference among samples (one-way ANOVA; Tukey HSD test; *p* ≤ 0.05). Volume and specific volume are expressed in mL and mL/g, respectively. Crumb firmness is expressed in N.

The addition of sorghum sprouted for 36 h and longer resulted in a higher volume and specific volume of bread (~12%), compared to bread from wheat flour alone (610 mL and 2.30 mL/g, respectively; data not shown) and the CTRL sample (Figure 6).

The positive effects of sprouting on crumb firmness were evident when sorghum sprouted from 36 h was used. In particular, the lowest firmness was observed in the 96 h-enriched bread (Figure 6). The replacement of wheat flour with sorghum decreased luminosity (up to ~26% for the 36 h sample) and increased the redness of crumb (up to ~100% for the 72 h sample). Moreover, using sprouted sorghum caused a slight decrease in crumb yellowness (up to ~10% for the 72 h and 96 h sample).

**Figure 7:**
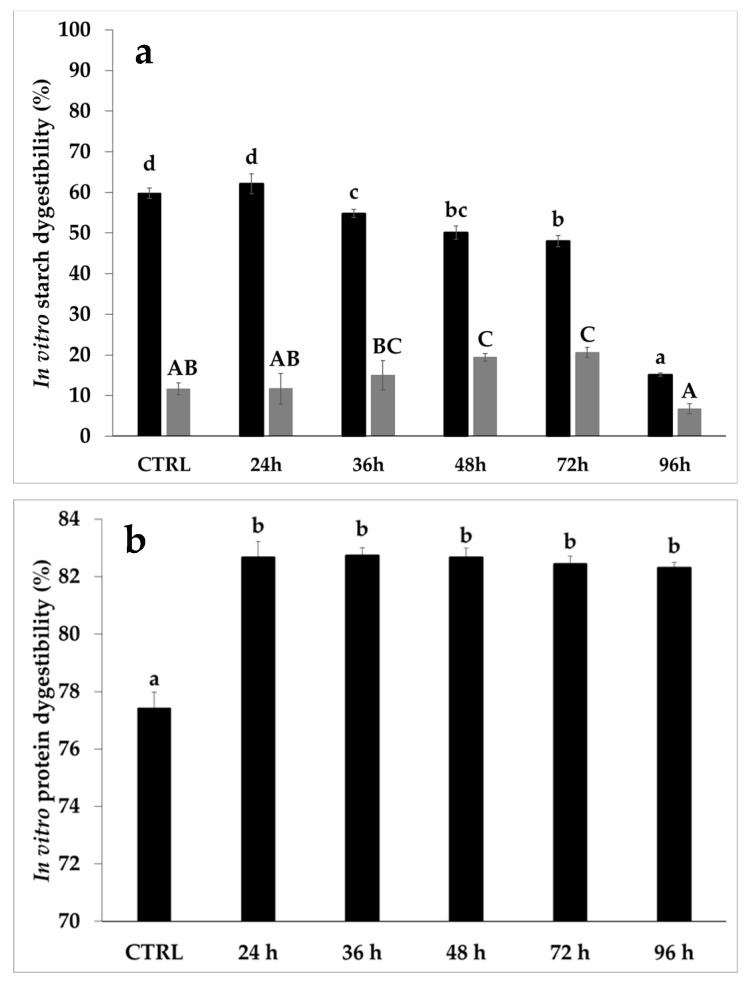
Rapidly (black bars; RDS) and slowly (gray bars; SDS) digestible starch (**a**), and protein digestibility (**b**) of wheat bread containing 20% unsprouted (CTRL) or sprouted sorghum at different times (24 h, 36 h, 48 h, 72 h, and 96 h). Different letters (lower case for rapidly digestible starch and protein digestibility; uppercase for slowly digestible starch) indicate a significant difference among samples (one-way ANOVA; Tukey HSD test; *p* ≤ 0.05).

As regards in vitro digestibility, the rapid digestible starch (RDS) significantly decreased from 36 h (~9%) up to 96 h (~75%) of sprouting. Unlike RDS, no difference was observed between CTRL and 96 h-enriched bread in terms of slowly digestible starch (SDS) (Figure 7a).

These results partly agreed with data reported by Swieca et al. [47], when a commercial wheat flour was replaced with 20% of flour from wheat sprouted for 96 h. These authors related the decrease in RDS to the increase in the resistant starch fraction and/or in the polyphenol content of the sprouted material [47]. Interestingly, the trend followed by the SDS fraction of 96 h-enriched bread was not the same as other samples, likely related to the different bread structure; however, this aspect needs to be further investigated.

Concerning the in vitro protein digestibility, sprouting caused an increase in this index, regardless of sprouting time (Figure 7b).

### 3.6. Principal Component Analysis (PCA)

**Figure 8:**
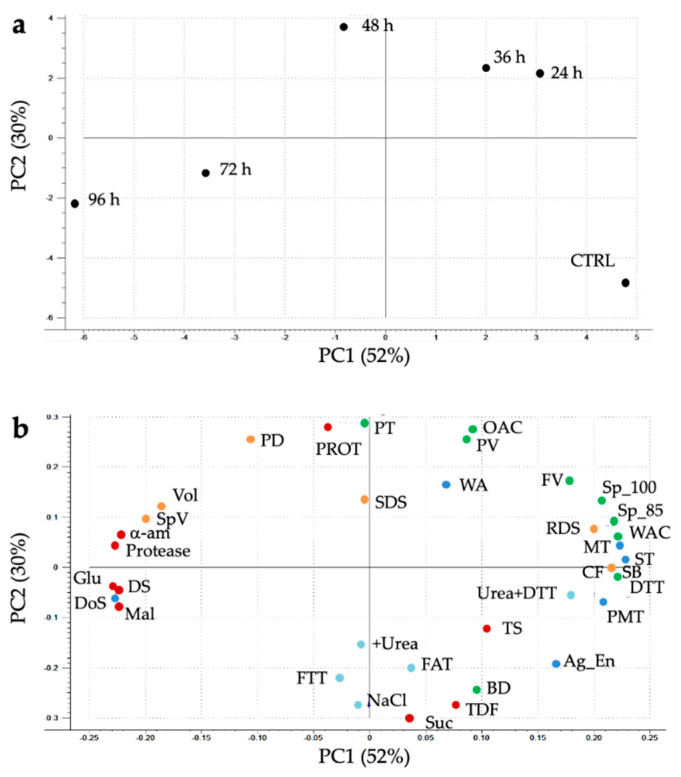
Score (**a**) and Loading (**b**) plots for Principal Component Analysis on chemical composition and enzymatic activities, protein solubility and thiols, gluten aggregation, mixing properties, and bread properties. α-am, α-amylase activity; DS, Damaged Starch; Glu, D-glucose; Mal, Maltose; Prot, Protein; Suc, Sucrose; TDF, Total Dietary Fiber; TS, Total Starch. Protein solubility: DTT, Dithiothreitol. Thiols: FAT, Free Accessible Thiols; FFT, Free Total Thiols. Functional properties: BD, Breakdown; FV, Final Viscosity; OAC, Oil Absorption Capacity; PT, Peak Temperature; PV, Peak Viscosity; SB, Setback; Sp_85, Swelling Power at 85 °C; Sp_100, Swelling Power at 100 °C WAC, Water Absorption Capacity. Gluten aggregation properties: PMT, Peak Maximum Time; MT, Maximum Torque; Ag_En, Aggregation Energy. Mixing properties: DDT, Dough Development Time; DoS, Degree of Softening; ST, Stability; WA: Water Absorption. Bread properties: CF, Crumb Firmness; PD, Protein Digestibility; RDS, Rapidly Digestible Starch; SDS, Slowly Digestible Starch; SpV, Specific Volume; Vol, Bread Volume.

The results of PCA reported the distribution of samples in accordance with chemical composition, enzymatic activities, protein solubility, thiols, gluten aggregation kinetics, mixing, and bread properties (Figure 8). The scores plot—which described about 82% of the variability of the data (PC1: ~52%; PC2: ~30%)—highlighted a separation of samples based on sprouting duration (Figure 8a). Indeed, the CTRL sample is in the bottom right corner, assuming highly positive and negative values for PC1 and PC2, respectively. The 24 h- and 36 h-sprouted samples are in upper right corner assuming positive values for both PC1 and PC2, while 48 h-sprouted sample is in upper left corner assuming the highest value of PC2; 72 h- and 96 h-sprouted samples are in the bottom left corner, tending to the negative values of both PC1 and PC2.

Furthermore, the loading plot identified the variables that determine sample grouping (Figure 8b). Most of the indices of chemical composition, enzymatic activities, functional properties, along with gluten aggregation and mixing properties, and bread characteristics determined the separation of samples along PC1, whereas indices mainly related to the protein features were responsible for the separation of sprouted samples along PC2.

## 4. Discussion

In recent times, sprouted grains, such as wheat [43,48,49], brown rice [50,51,52], quinoa [35,53,54,55], oat [55,56], finger millet [57], and pulses [57,58,59], have already been exploited in bread making to improve the features of composite bread. As regards sorghum, the effect of the sprouting process on chemical composition and/or functional properties has already been reported in several studies [4,14,15,16,17,18,19,21]. Until now, however, to the best of our knowledge, no studies elucidated the relation between flour functionality and bread-making performance of breads enriched in sorghum sprouted till 96 h.

Comprehending the effect of sprouting on starch and protein is important since these components are responsible for the properties of the final products, such as dough properties, bread staling, and digestibility. In this context, in the first part of this study the changes in the chemical and functional properties induced by sprouting duration were assessed. Then, the effect of such changes on dough and bread properties were studied. Specifically, sprouted sorghum was used at 20% level in a wheat-based formulation.

From a compositional standpoint, the greatest effect of sprouting time was observed for sugar and damaged starch content, as an effect of the increased α-amylase activity (Table 1). These results were supported also by PCA, since these indices were in upper and bottom left quadrants of the loading plot (Figure 8b), influencing the separation of samples according to the sprouting time along the PC1. In a recent study, the increase in damaged starch (i.e., starch susceptibility to α-amylase) was confirmed by observing some holes on the surface of starch granules of sprouted sorghum for 72 h [15]. In addition, increased proteolytic activity may also have played a role in increasing starch susceptibility to the α-amylases. Indeed, as hydrolysis of the proteins surrounding the starch granules proceeds, the starch granules are more easily accessible to the amylolytic enzymes. The hydrolysis of protein bodies during sprouting was confirmed by SDS-PAGE (Figure 3), in accordance with previous reports [15,60]. Consequently, the hydrolysis of protein bodies improved the in vitro starch and protein digestibility of bread (Figure 7). In addition, the hydrolysis of protein bodies at the initial stage of sprouting (24 h) make starch granules more available to gelatinize (Figure 1). The role of protein in decreasing the starch gelatinization ability is also supported by the different Sp of unsprouted and sprouted sorghum, found in this study (Table 2). In this regard, similar results were reported by Elkhalifa and Bernhardt [61], who demonstrated that sorghum Sp decreased by about 23% after 72 h of sprouting when evaluated at 85 °C, and about 39% at 100 °C.

Changes in protein structure might have increased the amount of lipophilic amino acids on the protein surface [14,23], with consequent increase in the flour ability to absorb oil (Table 2). The increased ability to absorb oil makes sprouted sorghum a suitable raw material for formulating products where the ability to absorb oil is crucial, such as in baby-foods and energy-dense snacks [62]. The decrease in the OAC shown by sorghum sprouted from 72 h (Table 2) might be due to the intense proteolytic activity (Table 1) leading to excessive protein hydrolysis, resulting in loss of OAC again. Elkhalifa and Bernhardt [14] observed an increase in sorghum OAC in the first 72 h of sprouting, and a decrease after 96 h. On the other hand, protein solubility was not strongly affected by proteolytic activity (Figure 2), likely due to the hydrolyzed products that remained associated with the original protein, as previously suggested by Suárez-Estrella et al. [63].

Moving to starch functionality, the changes in the protein–starch matrix, together with the increased enzymatic activities, resulted in a decrease in the pasting and gelation properties of sprouted samples for 36 h or longer (Table 2; Figure 1a), since the hydrolyzed starch is no longer able to form a rigid gel and simple sugars cannot absorb a high amount of water [64]. These results were in accordance with previous studies [1,4,65], and they were confirmed also by the PCA loading plot (Figure 8b), where the indices related to pasting and gelation properties were characterized by positive PC1 values, discriminating the CTRL sample from the sprouted ones. Moreover, the decrease in the retrogradation tendency of sprouted sorghum led to the production of bread with a softer crumb compared to CTRL-enriched bread (Figure 6), after one day of storage, regardless of crumb moisture (data not shown). Similar results were reported also when quinoa sprouted for 48 h was added at 20% replacement level to wheat bread [35].

Refined wheat flour was replaced with unsprouted and sprouted sorghum at 20% level, to produce sorghum-enriched bread. The gluten properties of the blends were evaluated both in slurry (i.e., GlutoPeak test) and dough (i.e., Farinograph test) systems, with different hydration and shear stress conditions. In this context, to the best of our knowledge, no study has previously evaluated the influence of sprouted sorghum on wheat flour properties, making it difficult to compare our results with the literature. The GlutoPeak test suggested a weakening of the gluten matrix when sprouted sorghum was used instead of CTRL (Table 3; Figure 4). This behavior might have been caused by the structural changes induced by sprouting on the sorghum components (i.e., protein and fiber) (Figure 2 and Figure 3), which might have interacted differently with wheat gluten proteins during gluten formation. In addition, the lower maximum torque of the sprouted samples compared with CTRL could be explained by the decrease in the accessible free thiols available to interact with wheat thiols to form a gluten matrix.

The results obtained by means of the GlutoPeak test were confirmed also in the dough system, with a decrease in both, the dough development time and stability, and the increase in the degree of softening (Table 3; Figure 5). Specifically, the worsening of mixing properties was related to sprouting time (Table 3), likely due to the increase in the proteolytic activity developed during the sprouting process (Table 1). Indeed, during farinograph test proteases are able to hydrolyze gluten proteins due to the long time of the test (20 min), resulting in a gluten weakening [66]. Similar results were also reported when 48 h-sprouted quinoa was used at 20% replacement level [35]. Despite the worsening in gluten aggregation and dough mixing properties, the dough was able to resist the stress during baking and did not collapse, except for the 96 h-enriched bread sample likely due to its high proteolytic activity and consequent higher gluten weakening. In fact, it was characterized by a lower height compared to samples sprouted between 36 and 72 h (data not shown). The increase in bread volume might also be related to the increase in simple sugars (Table 1), available for yeasts to produce CO_2_ during leavening [43,48,49]. Improvements in bread characteristics were also reported in previous studies carried out on sprouted common [48,49] and durum [43] wheat, and quinoa [35].

Finally, regarding starch digestibility, the increase in SDS (Figure 7a) in samples starting from 36 h of sprouting (Figure 7a) was associated with the increase in resistant starch (data not shown). In this context, there are controversial opinions in the literature on the sprouting effect on the in vitro starch digestibility of bread [22]. For instance, an increase in SDS fraction was observed in wheat bread enriched in sprouted wheat [49], while a decrease in SDS was measured in sprouted brown rice-based bread [67]. Different results might be explained by different analytical methods, as well as sprouting and bread-making conditions used.

In conclusion, the present research highlighted the relationship between chemical-functional properties induced by sprouting and bread properties. Specifically, sprouting resulted in the most intense changes in the functional properties starting from 36 to 48 h of the process, although increased hydrolytic activity occurred as early as 24 h of sprouting. As regards bread, by using sorghum sprouted between 36 and 72 h in a composite flour (20% replacement level) it was possible to obtain a bread with increased specific volume and decreased crumb firmness, stimulating the possibility of using sprouted sorghum in baked goods. Therefore, sprouting could represent an interesting strategy to fully exploit the potential of sorghum to be used in cereal-based products and ensure wide consumer attraction for this sustainable crop. Further studies will address the relationship between flour functionality and starch and protein structure, as affected by sprouting process.

## Figures and Tables

**Table 1:** Chemical composition and enzymatic activities of unsprouted (control-CTRL) and sprouted sorghum at different times (24 h, 36 h, 48 h, 72 h, and 96 h).

	CTRL	24 h	36 h	48 h	72 h	96 h
Total starch	77.5 ± 1.9 ^b^	73.2 ± 1.2 ^a^	75.0 ± 2.1 ^ab^	75.2 ± 2.5 ^ab^	73.7 ± 0.4 ^a^	74.3 ± 1.4 ^ab^
Damaged starch	9.4 ± 0.5 ^a^	9.6 ± 0.2 ^a^	9.6 ± 0.3 ^a^	11.5 ± 0.3 ^b^	12.7 ± 0.5 ^c^	13.6 ± 0.3 ^d^
Maltose	0.2 ± 0.1 ^a^	0.12 ± 0.03 ^a^	0.54 ± 0.09 ^b^	1.31 ± 0.03 ^c^	2.23 ± 0.03 ^d^	3.5 ± 0.3 ^e^
Sucrose	0.64 ± 0.05 ^d^	0.25 ± 0.05 ^a^	0.24 ± 0.01 ^a^	0.22 ± 0.08 ^a^	0.36 ± 0.05 ^b^	0.45 ± 0.03 ^c^
D-glucose	0.24 ± 0.01 ^a^	0.25 ± 0.01 ^a^	0.29 ± 0.08 ^a^	0.47 ± 0.02 ^b^	0.58 ± 0.01 ^c^	0.70 ± 0.01 ^d^
Protein	8.8 ± 0.1 ^a^	9.0 ± 0.1 ^ab^	9.1 ± 0.1 ^b^	9.1 ± 0.1 ^b^	9.0 ± 0.1 ^ab^	8.9 ± 0.1 ^ab^
Total fiber	7.2 ± 0.2 ^a^	6.9 ± 0.4 ^a^	6.8 ± 0.5 ^a^	6.7 ± 0.2 ^a^	7.0 ± 0.5 ^a^	6.9 ± 0.1 ^a^
Insoluble	82	78	85	82	89	90
Soluble	18	22	15	18	11	10
α-amylase	0.07 ± 0.02 ^a^	2.7 ± 0.6 ^b^	3.0 ± 0.4 ^bc^	3.7 ± 0.4 ^c^	4.5 ± 0.4 ^d^	6.2 ± 0.6 ^e^
Protease	1.3 ± 0.1 ^a^	2.5 ± 0.1 ^b^	2.7 ± 0.1 ^bc^	3.0 ± 0.1 ^c^	3.9 ± 0.2 ^d^	4.3 ± 0.2 ^e^

Different letters in the same row indicate a significant difference among samples (one-way ANOVA; Tukey HSD test; *p* ≤ 0.05). Compositional data are expressed as g/100g of flour d.b. Insoluble and soluble dietary fiber were expressed as g/100g of total dietary fiber. Damaged starch is expressed as g/100g of total starch. α-amylase and proteolytic activities are expressed as Ceralpha Units (CU/g flour d.b.), and as the activity/g of flour d.b., respectively.

**Table 2:** Functional properties of unsprouted (control, CTRL) and sprouted sorghum at different times (24 h, 36 h, 48 h, 72 h, and 96 h).

	CTRL	24 h	36 h	48 h	72 h	96 h
Hydration Properties						
WAC	1.50 ± 0.01 ^d^	1.48 ± 0.03 ^cd^	1.50 ± 0.01 ^d^	1.42 ± 0.03 ^bc^	1.38 ± 0.04 ^ab^	1.33 ± 0.02 ^a^
OAC	1.07 ± 0.02 ^a^	1.28 ± 0.02 ^b^	1.29 ± 0.04 ^b^	1.31 ± 0.04 ^cd^	1.08 ± 0.05 ^a^	1.03 ± 0.03 ^a^
Swelling Power (Sp)						
85 °C	7.2 ± 0.4 ^c^	6.7 ± 0.1 ^bc^	6.3 ± 0.2 ^bc^	6.3 ± 0.2 ^bc^	6.4 ± 0.1 ^bc^	5.4 ± 0.3 ^a^
						
100 °C	10.2 ± 0.1 ^bc^	10.8 ± 0.6 ^bc^	10.2 ± 0.5 ^bc^	9.7 ± 0.2 ^c^	5.7 ± 0.4 ^b^	3.5 ± 0.4 ^a^
Pasting properties in water						
Viscosity peak	1858 ± 21 ^e^	2131 ± 7 ^f^	1593 ± 44 ^d^	945 ± 4 ^c^	306 ± 11 ^b^	187 ± 1 ^a^
Peak temperature	77.8 ± 0.5 ^a^	81.2 ± 0.6 ^c^	79.3 ± 0.4 ^b^	78.0 ± 0.5 ^ab^	78.0 ± 0.5 ^ab^	77.5 ± 0.1 ^a^
Breakdown	739 ± 8 ^d^	706 ± 10 ^c^	826 ± 11 ^e^	720 ± 6 ^cd^	276 ± 13 ^b^	166 ± 2 ^a^
Final viscosity	2392 ± 75 ^d^	2509 ± 65 ^d^	1606 ± 67 ^c^	602 ± 22 ^b^	70 ± 2 ^a^	43 ± 1 ^a^
Setback	1273 ± 58 ^e^	1084 ± 55 ^d^	838 ± 35 ^c^	378 ± 14 ^b^	40 ± 1 ^a^	23 ± 1 ^a^

Different letters in the same row indicate a significant difference among samples (one-way ANOVA; Tukey HSD test; *p* ≤ 0.05). WAC and OAC are expressed as g/g of flour d.b. Sp is expressed as g/100g of sample d.b. Viscosity peak, breakdown, final viscosity and setback are reported in cP, while peak temperature in °C. WAC: Water Absorption Capacity; OAC: Oil Absorption Capacity.

**Table 3:** Gluten aggregation and mixing properties of wheat flour containing 20% unsprouted (control, CTRL) or sprouted sorghum at different times (24 h, 36 h, 48 h, 72 h, and 96 h).

	CTRL	24 h	36 h	48 h	72 h	96 h
*Gluten aggregation properties*						
Maximum torque	42.9 ± 1.0 ^b^	43.5 ± 1.5 ^b^	43.0 ± 1.3 ^b^	40.3 ± 1.2 ^ab^	39.5 ± 0.1 ^a^	37.6 ± 1.5 ^a^
Peak maximum time	217 ± 14 ^c^	180 ± 8 ^b^	166 ± 5 ^b^	167 ± 6 ^b^	125 ± 3 ^a^	135 ± 6 ^a^
Aggregation energy	1063 ± 8 ^c^	1075 ± 26 ^c^	1073 ± 22 ^c^	1029 ± 15 ^bc^	978 ± 5 ^ab^	935 ± 21 ^a^
*Mixing properties*						
Water absorption	61.6 ± 0.3 ^b^	61.7 ± 0.3 ^b^	61.5 ± 0.2 ^b^	61.9 ± 0.1 ^b^	61.6 ± 0.3 ^b^	61.4 ± 0.2 ^b^
Dough development time	3.0 ± 1.0 ^b^	3.0 ± 0.1 ^b^	2.7 ± 0.2 ^ab^	2.1 ± 0.2 ^ab^	2.0 ± 0.1 ^ab^	1.9 ± 0.2 ^a^
Stability	9.5 ± 0.1 ^cd^	9.0 ± 0.6 ^cd^	7.3 ± 0.4 ^c^	5.3 ± 0.4 ^b^	2.9 ± 0.6 ^a^	2.2 ± 0.3 ^a^
Degree of softening	57 ± 4 ^a^	62 ± 6 ^a^	84 ± 1 ^b^	108 ± 1 ^c^	167 ± 8 ^d^	191 ± 5 ^e^

Different letters in the same row indicate a significant difference among samples (one-way ANOVA; Tukey HSD test; *p* ≤ 0.05). Maximum torque is expressed in GlutoPeak Units (GPU); peak maximum time is expressed in s; aggregation energy is expressed in GlutoPeak Equivalent (GPE); water absorption is expressed in g/100g of flour d.b.; dough development time and stability are expressed in min; degree of softening is reported in Farinograph Units (FU).

## Data Availability

Not applicable.

## Abbreviations

α-am	α-amylase activity
Ag_En	Aggregation Energy
ANOVA	Analysis of Variance
BD	Breakdown
CF	Crumb Firmness
DDT	Dough Development Time
DoS	Degree of Softening
DS	Damaged Starch
DTT	Dithiothreitol
FAT	Free Accessible Thiols
FFT	Free Total Thiols
FU	Farinograph Units
FV	Final Viscosity
Glu	D-glucose
GPU	GlutoPeak Units
Mal	Maltose
MT	Maximum Torque
OAC	Oil Absorption Capacity
PD	Protein Digestibility
PMT	Peak Maximum Time
Prot	Protein
PT	Peak Temperature
PV	Peak Viscosity
RDS	Rapidly Digestible Starch
RVA	Rapid Visco Analyzer
SB	Setback
SDS	Slowly Digestible Starch
SDS-PAG	Sodium Dodecyl Sulfate
Sp	Swelling power
SpV	Specific Volume
ST	Stability
Suc	Sucrose
TDF	Total Dietary Fiber
TS	Total Starch
Vol	Volume
WA	Water Absorption
WAC	Water Absorption Capacity
